# Retraction: KCTD1 Suppresses Canonical Wnt Signaling Pathway by Enhancing β-catenin Degradation

**DOI:** 10.1371/journal.pone.0268604

**Published:** 2022-05-12

**Authors:** The PLOS ONE Editors

Following the publication of this article [1], concerns were raised regarding the results presented in multiple figures. Specifically,

- In Fig 4C, there appear to be repetitive elements in the background within and between lanes 5–10 when color levels are adjusted.
- In Fig 5B, there appear to be repetitive elements in the background of lanes 2–3 when color levels are adjusted.
- In Figs 7B and 8C, the following bands appear similar:
  ○ Lanes 1 and 2 of the β-catenin panel in Fig 7B and the β-catenin panel in Fig 8C flipped vertically.
  ○ Lanes 1 and 2 of the KCTD1 panel in Fig 7B and the KCTD1 panel in Fig 8C.
  ○ Lanes 1 and 2 of the β-actin panel in Fig 7B and the β-actin panel in Fig 8C flipped vertically.

The corresponding author acknowledged that Figs 4C and 5B contain repetitive elements in the background and stated that the figures were adjusted in error. They provided raw image data underlying Figs 4C and 5B and repeat experiments. The raw data did not resolve the concerns regarding repetitive elements which question the integrity of these data, and the journal does not consider alternative experiment data to be sufficient to address these concerns.

The corresponding author stated that the results in lanes 1–2 of Fig 7B and the results in Fig 8C represent the same experimental conditions. Furthermore, the corresponding author clarified that the β-catenin and β-actin blots in Fig 8C were inadvertently flipped across the horizontal axes during figure preparation.

In light of the concerns affecting Figs 4C and 5B that question the integrity of these data, the *PLOS ONE* Editors retract this article.

XL, CC, FW, WH, and XD did not agree with the retraction and stand by the article’s findings. All other authors either did not respond directly or could not be reached.
